# Optimizing Neurobehavioral Assessment for Patients with Disorders of Consciousness: Proposal of a Comprehensive Pre-Assessment Checklist for Clinicians

**DOI:** 10.3390/brainsci15010071

**Published:** 2025-01-15

**Authors:** Kristen Keech, Caroline Schnakers, Brooke Murtaugh, Katherine O’Brien, Beth Slomine, Marie-Michèle Briand, Rita Formisano, Aurore Thibaut, Anna Estraneo, Enrique Noé, Olivia Gosseries, Liliana da Conceição Teixeira

**Affiliations:** 1 The Queen’s Medical Center, Honolulu, HI 96813, USA; 2 Doctor of Occupational Therapy Program, Hawaii Pacific University, Honolulu, HI 96813, USA; 3 Research Institute, Casa Colina Hospital and Centers for Healthcare, Pomona, CA 91767, USA; cschnakers@casacolina.org; 4 Department of Rehabilitation Programs, Madonna Rehabilitation Hospitals, Lincoln, NE 68506, USA; 5 TIRR Memorial Hermann, Houston, TX 77030, USA; 6 Department of Physical Medicine & Rehabilitation, McGovern Medical School, The University of Texas Health Science Center at Houston, Houston, TX 77030, USA; 7 Brain Injury Clinical Research Center, Kennedy Krieger Institute, Baltimore, MD 21205, USA; 8 Department of Psychiatry and Behavioral Sciences, Johns Hopkins University School of Medicine, Baltimore, MD 21224, USA; 9 Physical Medicine and Rehabilitation Department, Hôpital du Sacré-Coeur de Montréal, Montreal, QC H4J 1C5, Canada; marie-michele.briand.med@ssss.gouv.qc.ca; 10 IRCCS Santa Lucia Foundation, 00179 Rome, Italy; r.formisano@hsantalucia.it; 11 Coma Science Group, GIGA-Consciousness, University of Liège, 4000 Liège, Belgium; 12 Centre du Cerveau, University Hospital of Liège, 4000 Liège, Belgium; 13 IRCCS, Don Gnocchi Foundation, 50143 Florence, Italy; 14 IRENEA Instituto de Rehabilitación Neurológica, Fundación Hospitales Vithas, 46011 Valencia, Spain; enoe@comv.es; 15 RISE-Health, Center for Translational Health and Medical Biotechnology Research (TBIO), ESS, Polytechnic of Porto, R. Dr. António Bernardino de Almeida, 400, 4200-072 Porto, Portugal; teixeiraliliana@hotmail.com

**Keywords:** assessment, disorders of consciousness, brain injury, survey

## Abstract

Background: Clinicians are challenged by the ambiguity and uncertainty in assessing level of consciousness in individuals with disorder of consciousness (DoC). There are numerous challenges to valid and reliable neurobehavioral assessment and classification of DoC due to multiple environmental and patient-related biases including behavioral fluctuation and confounding or co-occurring medical conditions. Addressing these biases could impact accuracy of assessment and is an important aspect of the DoC assessment process. Methods: A pre-assessment checklist was developed by a group of interdisciplinary DoC clinical experts and researchers based on the existing literature, current validated tools, and expert opinions. Once finalized, the checklist was electronically distributed to clinicians with a range of experience in neurobehavioral assessment with DoC. Respondents were asked to use the checklist prior to completing a neurobehavioral assessment. A survey was also provided to respondents to obtain feedback regarding checklist feasibility and utility in optimizing the behavioral assessments. Results: Thirty-three clinicians completed the survey after using the checklist. Over half of the respondents were a combination of physicians, neuropsychologists, and physical therapists. All respondents served the adult DoC population and 42% percent had over ten years of clinical experience. Eighty percent reported they found the format of the checklist useful and easy to use. All respondents reported the checklist was relevant to preparing for behavioral assessment in the DoC population. Eighty-four percent reported they would recommend the use of the tool to other clinicians. Conclusions: The use of a pre-assessment checklist was found to be feasible and efficacious in increasing interdisciplinary clinician’s ability to optimize the patient and environment in preparation for neurobehavioral assessment. Initial results of clinicians’ perception of the utility of a pre-assessment checklist were positive. However, further validation of the tool is needed with larger sample sizes to improve representation of clinical use across disciplines and care settings.

## 1. Introduction

After a severe acquired brain injury, patients can present an acute or prolonged disorders of consciousness (DoCs), which include coma, unresponsive wakefulness syndrome (UWS; previously known as vegetative state—VS) and minimally conscious state (MCS). Patients in UWS/VS open their eyes and demonstrate preserved reflexive functions but are not conscious, while MCS is characterized by the presence of inconsistent but discernible behavioral signs of consciousness (e.g., visual tracking, command following) [1,2,3]. Recently, MCS has been subdivided into more specific clinical entities including MCS- and MCS+, characterized by the absence or presence of response to a command, intelligible verbalization, and/or intentional communication [4]. Once a patient regains functional communication or purposeful use of objects on repeated assessments, they are identified as emerged from MCS (EMCS) [5].

The most common modality used for diagnosis of level of consciousness is bedside neurologic examination observing arousal and responsiveness [6,7]. Accurately assessing behavioral signs of consciousness in patients with DoC can be difficult due to a multitude of factors. These factors can include examiner experience, error, examiner bias, and patient and environmental factors [6,8,9]. Past evidence indicates that misdiagnosis rates for levels of consciousness in DoC are approximately 40%, often involving the erroneous classification of patients as being in a UWS when they are actually in an MCS [10]. Inaccurate diagnosis of DoC can have significant clinical and ethical implications for prognostic counseling, early withdrawal of life-sustaining treatment, and access to rehabilitation and post-acute care resources [11,12,13,14].

The American and European Academies of Neurology have published DoC guidelines and practice recommendations addressing assessment, diagnosis, prognosis, and treatment in the acute and subacute stages after a severe acquired brain injury [15,16]. These evidence-based recommendations include the use of valid and reliable neurobehavioral assessment to evaluate patient responses to various sensory stimuli (i.e., auditory, visual, motor, communication). The American Congress of Rehabilitation Medicine has recommended several behavioral scales for diagnosing DoCs, such as the Coma Recovery Scale-Revised (CRS-R), but also the Sensory Modality Assessment and Rehabilitation Technique (SMART), the Wessex Head Injury Matrix, Western Neuro Sensory Stimulation Profile (WHIM), the Sensory Stimulation Assessment Measure (SSAM), and the Disorders of Consciousness Scale (DOCS) [17]. However, the outcomes of these assessments are heavily influenced by proper implementation and the conditions under which the assessments are conducted [18,19].

The validity and reliability of the results from these assessment tools can be affected by both environmental factors and biases related to the patient. The patient’s positioning, underlying medical conditions, complications, and variables of spasticity, sensory deficits, and neuropsychological disorders such as aphasia can confound assessment by obscuring a patient’s ability to demonstrate consciousness [8,20,21]. Neglecting these factors can lead to misinterpretation of level of consciousness, potentially resulting in inappropriate treatment decisions. A checklist designed to optimize both the patient and the environment prior to conducting a bedside consciousness assessment could be a valuable tool for enhancing the accuracy and reliability of the scores and, therefore, of the diagnosis. Few existing scales, such as the SMART, include a checklist to guide clinicians to identify confounds and optimize the patient during the assessment [22]. However, many other consciousness assessment scales are utilized clinically outside of the SMART and none include a checklist to identify and potentially decrease such biases before the assessment occurs. Hence, this study aimed to evaluate the effectiveness of a newly developed pre-assessment checklist in facilitating valid and reliable neurobehavioral assessments of consciousness.

## 2. Methods

### 2.1. Study Design

(a) Checklist development. A group of 12 members from the Special Interest Group on DoC of the International Brain Injury Association (IBIA DoC-SIG) developed the checklist. The members of this group are international experts on DoC including physicians, neuropsychologists, occupational therapists, and physical therapists practicing in various locations mostly around the United States and Europe. The members were asked to review existing validated tools recommended by relevant professional association [17], as well as recent guidelines regarding the assessment of DoC [15,16]. The material was reviewed and discussed over recurrent virtual meetings (between April 2022 and July 2024) to guide the development and inclusion of the checklist items. A series of items were identified as essential for assessing the potential impact of patient- and environment-related biases on assessment results. Table 1 highlights the Category Domains of the Checklist. Pre-assessment checklist items included patient’s personal information, medical conditions and confounds, assessment location and scale used, patient and environmental considerations prior to starting the assessment, and patient testing position. The finalized checklist was three pages in length. A draft for the checklist was submitted to the group and a consensus among experts was reached for the final version (Supplemental Material S1)
(b) Survey development. A survey was created on Google forms by the same panel of experts to investigate clinicians’ perception of using the checklist. Survey items were agreed upon through panel consensus over additional virtual meetings. The final survey included nineteen questions to elicit feedback on the feasibility and use of the checklist (Supplemental Material S2).

### 2.2. Sample

The inclusion criteria for survey respondents were clinicians within the disciplines of occupational and physical therapy, speech language pathology, psychology, physicians, and nurses who serve the pediatric and adult DoC population and conduct bedside neurobehavioral assessment. Rehabilitation and allied health professionals were part of the inclusion criteria as it is the authors’ experience that these disciplines are most commonly involved in implementation of bedside neurobehavioral assessment such as the CRS-R, DOCS, SMART, and other assessments listed previously [9,23]. Previous studies report that standardized bedside neurobehavioral assessments such as the CRS-R are rarely utilized by physicians [24]. The Glasgow Coma Scale, which is a non-standardized clinical tool to identify coma and classify severity of brain injury, is the tool most commonly used by physicians [7].

Participation was voluntary and no incentives were offered. Respondents were provided the checklist electronically through convenience sampling and the snowball method of recruitment and distribution. Respondents were instructed to use the checklist at least once, prior to conducting their consciousness assessment (specific behavioral scales were not recommended and were decided upon by clinician preference). Participants were then instructed to complete the electronic survey and were provided with a link to the survey. Informed consent was obtained from participants who completed the survey. Respondents and responses were anonymous. Anonymized data were collected over a three-month period to account for variations in the volume of patients with DoC across different care settings.

### 2.3. Data Analysis

Using Microsoft Excel, descriptive analyses (proportion, frequency, percentage) were conducted on questions with dichotomous answers (yes/no) and on questions with a five-point Likert scale: strongly disagree (1), disagree (2), neutral (3), agree (4), and strongly agree (5). Qualitative analysis was used to address the open-ended questions of the survey to identify themes. The included figures were also created using Microsoft Excel.

## 3. Results

### 3.1. Respondent Characteristics

**Figure 1:**
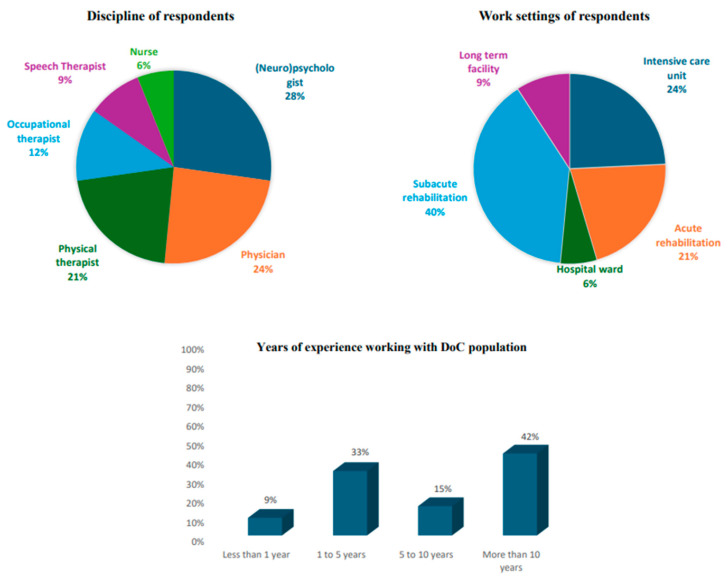
Characteristics of respondents; discipline, work setting, and years of experience of respondents.

Thirty-three surveys were completed at the end of the three-month study timeframe. Respondents who completed the survey treated individuals in DoC across various settings (see Figure 1), with 51.5% working in an acute setting (intensive care unit, *n* = 8; acute rehabilitation unit, *n* = 7; hospital ward, *n* = 2), 39.4% of respondents working in a subacute rehabilitation setting (*n* = 13), and 9.1% respondents working in a long-term care setting (*n* = 3).

Respondents were (neuro)psychologists (*n* = 9; 27.3%), physicians (*n* = 8; 24.2%), physical therapists (*n* = 7; 21.2%), occupational therapists (*n* = 4; 12.1%), speech therapists (*n* = 3; 9.1%), and nurses (*n* = 2; 6.1%) (see Figure 1). The majority of respondents (*n* = 24; 72.7%) reported working exclusively with the adult DoC population while only 6.1% (*n* = 2) were working with the pediatric population, and the remaining 21.2% (*n* = 7) indicated working with both groups.

More than half of the respondents had more than 5 years of clinical experience in the care of patients with DoC (See Figure 1). Five respondents reported 5–10 years and fourteen reported 10 or more years of clinical experience. Most respondents (78.8%; *n* = 26) reported using the CRS-R as the consciousness assessment of choice. Among these respondents, 30.8% of clinicians used another scale in combination with the CRS-R (*n* = 8). Finally, 21.2% used other scales than the CRS-R (*n* = 7). Other scales included the Coma Recovery Scale-Revised For Accelerated Standardized Testing [25] (CRS-R FAST; *n* = 1), the Simplified Evaluation of CONsciousness Disorders [22] (SECONDs; *n* = 4), the DOCS [26] (*n* = 1), the Disability Rating Scale [27] (DRS; *n* = 2), the WHIM [28] (*n* = 1), the Nociception Coma Scale-Revised [29] (NCS-R; *n* = 1), the Glasgow Outcome Scale-Extended [30] (GOS-E; *n* = 1), the Coma/Near Coma Scale [31] (CNCs; *n* = 2), the Levels of Cognitive Functioning Assessment Scale [32] (LOSFAS; *n* = 2), and the Agitated Behavior Scale [33] (ABS; *n* = 1).

### 3.2. Qualitative Data

A majority of responders agreed that they liked the format of the checklist (78.5%) and found it easy to use (87.9%). All respondents agreed that the checklist was relevant to the assessment of DoC population. Most respondents also agreed that the checklist could help in their practice (82%) and would recommend it to a colleague (85%) while most disagreed with noting inconsistencies when using it (85%) (See Table 2).

When asked how to improve the tool, several themes arose regarding areas for improvement. A recurrent theme from respondents recommended consolidating the checklist to 1–2 pages (*n* = 7). Respondents elaborated that the tool could be more concise to decrease administration time, particularly in the hospital setting where time at the bedside can be limited. Additionally, some respondents indicated that the checklist could be made more visually compact and easier to use by eliminating redundancies. However, they did not elaborate or provide specific examples. One respondent added that some descriptors were too long and difficult to interpret, which may add time for users to administer the checklist, especially considering the purpose of the checklist is for pre-assessment. Yet again, there was no elaboration beyond this statement. Many respondents (*n* = 22) felt the tool was comprehensive and made them consider all possible patient and environmental factors which may have been otherwise missed. Some felt the tool would be useful when training colleagues with less experience (*n* = 5). Others appreciated the benefit of the checklist to promote a standardized pre-assessment approach within and across disciplines (*n* = 9). Finally, a small subset of respondents suggested the developers expand aspects of the tool. Suggested changes included adding space for documenting familiar stimuli as indicated by the patient’s family, additional details to document motor performance, clarifying when splints/braces cannot be removed due to orthopedic precautions, and adding a checkbox to “remove restraints.” (see Table 3).

Secondary analyses were conducted to compare responses regarding the ease of use and usefulness of the checklist based on respondents’ levels of expertise (years of experience) and profession (see Table 4). Overall, most respondents (>75%) considered the checklist both easy to use and helpful, regardless of their expertise or background. While a high agreement rate on its usefulness was observed (78.6%), respondents with more experience were more likely to find the checklist easy to use (92.9% compared to 78.6%). Clinicians with less experience noted that some descriptions were lengthy and challenging to interpret in real-time. Additionally, they found certain instructions were worded in ways that did not align clearly with the provided answers, creating confusion. Some respondents recommended alternate wording to improve clarity and acknowledged they expected their ability to interpret the checklist to improve with continued use. This can be correlated with previous literature that showed implementation and validity of neurobehavioral assessment and results can be impacted by level of clinical experience [8,9]. Notably, all (neuro)psychologists and speech therapists reported the checklist as helpful in their practice, with the majority (91.7%) also finding it easy to use. Among occupational and physical therapists, 78.6% indicated the tool was helpful, while the majority (85.7%) found the tool was easy to use. Finally, 80% of physicians and nurses found the tool helpful to their practice and nearly all (90%) reported the tool was easy to use.

## 4. Discussion

This study was conducted to determine the utility of a pre-assessment checklist to support clinicians in improving optimization of the patient factors, intentional consideration, and minimization of confounds to enhance the validity of results in neurobehavioral assessment for individuals with DoC. The overarching objective of the checklist and subsequent survey to evaluate its utility was to provide clinicians a tool to systematically address patient and environmental factors that could impact the accuracy of consciousness assessment implementation. The consequences of inaccurate diagnosis of consciousness, specifically those of coma or vegetative state, such as withdrawal of life-sustaining treatment, can be dire [10,13,34]. Using a checklist to improve consciousness assessment accuracy can decrease this risk, as well as improve access to post-acute rehabilitation [19].

Thirty-three clinicians utilized the checklist and completed the survey. Over 50% practiced within an inpatient setting and had five or more years of experience. This suggests the feedback on checklist was provided by experienced clinicians, lending credibility to the tool’s perceived utility. Physicians, psychologists, and physical therapists comprise the majority of respondent disciplines. The CRS-R was the most utilized standardized neurobehavioral assessment tool, with 81.2% of respondents stating it is the assessment of choice. This aligns with the existing literature highlighting the CRS-R as the recommended tool and as being most widely used for evaluating patients with DoC [15,19].

All clinicians who utilized the checklist found the tool relevant and helpful in effectively optimizing the environment and patient prior to assessment. Additionally, respondents with less than five years of experience found the tool beneficial, particularly for guiding their pre-assessment preparation and potentially improving the reliability of assessment for novice clinicians. Respondents working in acute hospital settings provided suggestions for improvement, advocating for a more concise version with additional space for free-text comments. This highlights the need for flexibility in the design of clinical tools to meet the demands of fast-paced, high-pressure environments like acute care.

Qualitative feedback, outside the Likert scale, revealed several important themes. Respondents highlighted the checklist’s utility in training less experienced staff, offering a step-by-step guide to ensure thorough preparation for patient assessment. Second, they reported that the tool helped standardize their approach to pre-assessment, minimizing the risk of overlooking critical factors such as patient alertness, positioning, or environmental distractions. Additionally, the checklist facilitated clearer documentation by providing consistent language for clinicians to use when recording their assessment findings. These insights suggest that the checklist could serve not only as an immediate practical tool but also as an educational resource, enhancing the consistency and quality of care provided to DoC patients.

This study nevertheless has several limitations. There was a small sample size (*n* = 33) of respondents, limiting the generalizability of results to a larger cohort of clinicians and practice settings. A larger secondary globally diverse study on the use of the checklist is needed to increase the generalizability of results. The survey did not inquire about the training or competency level of the clinician in implementation of their chosen DoC assessment. Thus, the level of experience and expertise in bedside assessment, regardless of years of experience, is unknown, and this could have impacted perceived clinical use. The small sample size also limits further suggestions of modifications to the checklist that could be beneficial for clinical use. The authors also acknowledge that a small percentage of respondents reported using assessments that have not been specifically identified as specified measures of consciousness, including the DRS, GOSE, and ABS, which may impact the reported feasibility and utility of the checklist. A further limitation is that the checklist was mostly utilized with the assessment of the adult population, leaving room for further exploration of the utility with the pediatric DoC population. These limitations identify future research opportunities, including investigation of whether use of a checklist impacts the trajectory of consciousness assessment scores and can enhance diagnostic accuracy.

## 5. Conclusions

This is the first known study examining effectiveness of a novel pre-assessment checklist to optimize the assessment of patients in DoC and to mitigate potential confounds that could negatively impact the reliability and validity of assessment results. Our survey findings suggest that the use of this checklist is a feasible solution to enhance the consistency and accuracy of DoC assessment across the interdisciplinary team. Moreover, the tool could enhance clinician training and standardize documentation of patient and environmental factors during testing, further supporting clinical utility. Overall, the study suggests this checklist can be valuable in improving assessment of consciousness by ensuring a structured, standardized, and thorough pre-assessment process. Such an approach holds promise for advancing best practices in the field and ensuring more accurate diagnostic and prognostic outcomes for patients with DoC.

## Figures and Tables

**Table 1:** Checklist Category Domains.

Category	Included Key Items	Purpose
Patient Information	Name/Preferred Name Hand dominance Native Language Cultural/Religious considerations	To ensure an individualized approach to assessment
Medical Conditions and Confounds	Premorbid medical conditions Ventilator/O_2_ support Hearing or visual aids Motor Cognitive Sensorial Neurologic Behavior	To prompt the use to consider factors that may impact patient’s performance.
Location and assessment used	Assessment scale used Location	To identify assessment and potential variations in performance based on location.
Patient and Environmental Considerations	Temperature, lighting Sedating medications Acute fever/complication Family present Affective responses	To document environmental factors that may influence patient performance and determine optimal testing environment for future assessment.
Patient testing position	Supine Sitting Supported Standing	To accurately capture testing position and identify optimal position for arousal.

**Table 2:** Respondents’ feedback on the checklist.

	Strongly Agree	Agree	Neutral	Disagree	Strongly Disagree
**I like the format of the tool**	24%	54.5%	18.1%	0%	0%
**The tool was easy to use**	45.5%	42.4%	12.1%	0%	0%
**The tool can help me in my practice**	33.3%	48.5%	15.2%	3.0%	0%
**I would suggest this tool to colleagues/students**	51.5%	33.3%	15.2%	0%	0%
**I notice inconsistencies as I use the tool**	3.0%	0%	12.1%	18.2%	66.7%

**Table 3:** Qualitative themes of survey responses.

Theme	Description
Comprehensive	Respondents reported the checklist included most if not all considerations prior to an assessment.
Easy to use	Respondents indicated the checklist was logical, straight forward, and easy to use prior to completing an assessment. The check boxes made it easy to use.
Consolidation	Respondents indicated that some descriptions were too long and felt the checklist would be more efficient and accessible to complete if more compact.
Consideration of patient and environmental factors	Respondents noted that the checklist provides clinicians with a guide to promote optimizing of the patient and testing environment to establish a baseline and track recovery.
Standardizes approach	Respondents noted that the checklist is anticipated to increase consistency across the various disciplines of the team.
Valuable for training	Respondents reported that this tool would be beneficial in training clinicians with less experience in optimal assessment of patients with DoC.

**Table 4:** Level of agreement (agree to strongly agree) according to the respondents’ level of expertise and profession.

	Ease of Use	Helpful in Practice
**Expertise**		
<5 years (*n* = 14)	78.6%	78.6%
>5 years (*n* = 19)	92.9%	78.6%
**Profession**		
ST/Psych (*n* = 12)	91.7%	100.0%
OT/PT (*n* = 11)	85.7%	78.6%
Dr/Nurse (*n* = 10)	90.0%	80.0%

Legend: ST = speech therapist, Psych = (neuro)psychologist, OT = occupational therapist, PT = physical therapist, Dr = physician.

## Data Availability

The raw data supporting the conclusions of this article will be made available by authors on request.

## Supplementary Materials

The following supporting information can be downloaded at: https://www.mdpi.com/article/10.3390/brainsci15010071/s1, Supplemental Material S1: Neurobehavioral Checklist; Supplemental Material S2: Checklist Survey.
