# Supplementary material for: Optimizing Neurobehavioral Assessment for Patients with Disorders of Consciousness: Proposal of a Comprehensive Pre-Assessment Checklist for Clinicians
Source: Brain Sci. 2025 Jan 15;15(1):71. doi: 10.3390/brainsci15010071 (PMC11763646; doi:10.3390/brainsci15010071)
Supplement: Supplementary file 1 [file brainsci-15-00071-s001.zip › Supplementalmaterial2.Satifaction Survey.pdf]

Satisfaction survey

Behavioral assessment of patients with disorders of consciousness (DoC) is challenging. With experience, assessors came up with tips, and habits. In order to share these tips with clinicians less experienced, a group of experts developed a checklist to promote awareness of the multiple details to look for before starting a behavioral assessment.

The goal of this survey is to improve the tool developed to make it the most useful and efficient. Your feedback is very important because it will allow us to create the best version possible. Please be honest and share your observations with us.

By agreeing in the participation of this project, you are accepting to share anonymized data that could be used in a scientific publication.

Please let us know, by your answer to the first question, if you agree.

c.schnakers@gmail.com Switch account

Not shared

* Indicates required question

Do you agree to participate in this project and survey? *

- ○ Yes
- ○ No

Satisfaction survey

c.schnakers@gmail.com Switch account

Not shared

* Indicates required question

About the assessor

What is your professional background? *

- Neuropsychologist
- Nurse
- Occupational therapist
- Speech Therapist
- Physical therapist
- Physician
- Psychologist
- Other: ______

How experienced are you in the care of patients with DoC? *

- Less than 1 year
- 1 to 5 years
- 5 to 10 years
- More than 10 years

Do you have experience with :

- Adults population
- Pediatric population
- Both

If you use a behavior scale to assess patients with DoC, which scale do you use the most? *

Your answer

Do you have experience with :

- ◯ Adults population
- ◯ Pediatric population
- ◯ Both

If you use a behavior scale to assess patients with DoC, which scale do you use the most? *

Your answer

Is the named scale above relevant for both adult and pediatric DoC? *

- ◯ Yes
- ◯ No

On average, how many patients have you assessed in the last 12 months?

- ◯ Less than 5
- ◯ 5-10
- ◯ 10-20
- ◯ 20-30
- ◯ Greater than 30

In what setting did you use the checklist? *

- ☐ Intensive care unit
- ☐ Hospital ward
- ☐ Rehabilitation facility
- ☐ Long term facility
- ☐ Skilled nurse facility
- ☐ Other: ____________

Back Next Clear form

Satisfaction survey

c.schnakers@gmail.com Switch account

Not shared

* Indicates required question

Tool

For the next questions, answer according to your agreement with the proposed statement regarding the checklist tool: 1 strongly disagree to 5 strongly agree

I trialed the tool:

- 1 time
- 2 to 5 times
- More than 5 times

I like the format of the tool. *

- 1 (strongly disagree)
- 2
- 3
- 4
- 5 (strongly agree)

Can you comment about the format?

Your answer

The tool is easy to use. *

- 1 (strongly disagree)
- 2
- 3
- 4
- 5 (strongly agree)

Is the tool relevant for the patient(s) I assess?

- ◯ Yes
- ◯ No

The tool could help me in my practice. *

- ◯ 1 (strongly disagree)
- ◯ 2
- ◯ 3
- ◯ 4
- ◯ 5 (strongly agree)

Can you describe why?

Your answer

I would suggest this tool to colleagues/students? *

- ◯ 1 (strongly disagree)
- ◯ 2
- ◯ 3
- ◯ 4
- ◯ 5 (strongly agree)

I notice inconsistencies as I use it. *

- ◯ 1 (strongly disagree)
- ◯ 2
- ◯ 3
- ◯ 4
- ◯ 5 (strongly agree)

Back Next Clear form

Satisfaction survey

c.schnakers@gmail.com Switch account

Not shared

* Indicates required question

For the next 2 questions, descriptive answers are welcome.

What did you like in the tool? *

Your answer

Do you have any suggestions to improve the tool? *

Your answer

Back Submit Clear form
