# Supplementary material for: Optimizing Neurobehavioral Assessment for Patients with Disorders of Consciousness: Proposal of a Comprehensive Pre-Assessment Checklist for Clinicians
Source: Brain Sci. 2025 Jan 15;15(1):71. doi: 10.3390/brainsci15010071 (PMC11763646; doi:10.3390/brainsci15010071)
Supplement: Supplementary file 1 [file brainsci-15-00071-s001.zip › Supplementalmatrial1.NeurobehavioralChecklist.pdf]

Assessor's name:________________________ Job Title:______________________________

Neurobehavioral Pre-Assessment Checklist

Name	
Preferred name/Nickname	
Date of Birth	
Hand Dominance	
Native Language	
Cultural/Religious considerations	

Medical Condition and Assistive Devices

	Yes	No	Comments/Concerns
Hearing Devices	☐	☐	
Glasses	☐	☐	
Orally Intubated	☐	☐	
Tracheostomy	☐	☐	
If tracheostomy, use of speaking valve	☐	☐	
Supplemental Oxygen	☐	☐	
Ventilatory support	☐	☐	
Metabolic disorders	☐	☐	
Please list any relevant premorbid medical history			

Please take the following conditions into consideration that may influence the neurobehavioral assessment results.

Motor	Cognitive	Sensorial	Neurological/ Neurosurgical	Behaviour
• Spinal cord injury<br>• Neuropathy/myopathy<br>• Tremor/Myoclonus<br>• Spasticity/retractions<br>• Fractures<br>• Others:	• Aphasia<br>• Apraxia<br>• Agnosia<br>• Others:	• Blindness<br>• Deafness<br>• Others:	• Uncontrolled seizures<br>• Active hydrocephalus<br>• Craniectomy<br>• Ptosis*<br>• Others:<br><br>**If suspected ptosis, consider manual eye opening*	• Agitation/aggressivity<br>• Paroxysmal sympathetic hyperactivity<br>• Suspected pain or discomfort<br>• Others:

Note: this checklist can be applied prior to any assessment scale

Assessor's name:________________________ Job Title:______________________________

Assessment		
Date	AM / PM	
Assessment scale used		
Hospital ward or Home (please specify)		

Prior to starting assessment, please consider:

	Yes	No	N/A	Comments
Best time for patient's responsiveness	☐	☐	☐	
Rest period prior to session	☐	☐	☐	
Family involvement/input/presence	☐	☐	☐	
Familiar stimuli identified	☐	☐	☐	
Location of assessment	☐	☐	☐	
"Do not disturb" sign up	☐	☐	☐	
Adequate Temperature	☐	☐	☐	
Adequate Lighting	☐	☐	☐	
Minimize noise *(music/tv off)*	☐	☐	☐	
Door closed/close curtains if not single room	☐	☐	☐	

Note: this checklist can be applied prior to any assessment scale

Assessor's name:________________________ Job Title:______________________________

Tracheal suctioning needs	☐	☐	☐	
Sedative meds administered prior to session	☐	☐	☐	
Acute illness/fever	☐	☐	☐	
Skin integrity	☐	☐	☐	
Patient's wakefulness *(i.e., eye opening; needed prompts to wake up)*	☐	☐	☐	
Remove splints/casts	☐	☐	☐	
Remove sheets from covering body	☐	☐	☐	
At least 1 min observation prior to session	☐	☐	☐	
Documented affective responses *(e.g. smile, grimace, etc)*	☐	☐	☐	

Patient Testing Position

(Reposition as needed to maintain optimal positioning, avoid discomfort, and avoid fatigue)

☐ Supine ☐ Supine with head of bed elevated (please specify degree of incline___)

☐ Sitting in wheelchair ☐ Sitting on the mat

☐ Supported standing ☐ Other:____

Note: this checklist can be applied prior to any assessment scale
